# Interleukin-6 and Type-I Collagen Production by Systemic Sclerosis Fibroblasts Are Differentially Regulated by Interleukin-17A in the Presence of Transforming Growth Factor-Beta 1

**DOI:** 10.3389/fimmu.2018.01865

**Published:** 2018-08-13

**Authors:** Aleksandra Maria Dufour, Montserrat Alvarez, Barbara Russo, Carlo Chizzolini

**Affiliations:** ^1^Department of Immunology & Allergy, University Hospital and School of Medicine, Geneva, Switzerland; ^2^Department of Pathology & Immunology, University Hospital and School of Medicine, Geneva, Switzerland

**Keywords:** systemic sclerosis, interleukin-17A, transforming growth factor-beta, interleukin-6, type-I collagen, monocyte chemotactic protein-1, fibrosis

## Abstract

Functional cytokine networks have been poorly characterized in systemic sclerosis (SSc). While interleukin-17A (IL-17A) is increased in SSc skin and other organs, its role is still debated, particularly considering fibrogenesis. We uncover here a dual function of IL-17A in the presence of transforming growth factor-β 1 (TGF-β), the master pro-fibrotic cytokine. In the one hand, we report an unexpected synergic activity resulting in enhanced production of IL-6 by dermal fibroblasts; in the other hand, a substantial inhibition of type I collagen (col-I) production. IL-17A or TGF-β enhanced the production of IL-6 by 8- to 16-folds when compared to control in healthy donors (HD) and SSc cultures. However, the joint presence of IL-17A and TGF-β resulted in robustly exuberant responses with levels of IL-6 up to 100-folds higher than those observed in untreated cells. Inhibition of NFκB signaling pathway preferentially inhibited the production of IL-6 driven by IL-17A in HD fibroblasts, while inhibition of PI3K preferentially inhibited the production of IL-6 driven by TGF-β. Interestingly, when p38 MAPK was inhibited, substantial reduction of IL-6 production was observed for both IL-17A and TGF-β. Consistently with the inhibition experiments, the combined stimulation of fibroblasts by IL-17A and TGF-β resulted in 1.8-fold increase in p38 MAPK phosphorylation (*P* = 0.025), when compared to levels of phosphorylated p38 MAPK induced by IL-17A alone. Furthermore, the enhanced phosphorylation of p38 MAPK in the joint presence of IL-17A and TGF-β was unique among the signaling molecules we examined. As expected, TGF-β induced SMAD2 phosphorylation and col-I production. However, in fibroblasts cultured in the joint presence of TGF-β and IL-17A, SMAD2 phosphorylation was decreased by 0.6-folds (*P* = 0.022) when compared to that induced by TGF-β alone. Remarkably, in this condition, the production of col-I and fibronectin was significantly decreased in both HD and SSc. Thus, IL-17A and TGF-β reciprocally influence each other effector functions in fibroblasts. Intracellular molecular switches may favor synergic or antagonistic activities, which are revealed by specific readouts. The implications of these data in the context of SSc are far reaching, particularly in terms of therapeutic approaches since IL-6, IL-17A, and TGF-β are all putative targets of treatment.

## Introduction

Systemic sclerosis (SSc) is a connective tissue disorder characterized by fibrosis of the skin and internal organs, vasculopathy, and dysregulated immuno-inflammatory responses. Fibrosis is a characteristic aspect of the disease, bears a high token of morbidity and mortality (1), and is caused by an excess of extracellular matrix (ECM) deposition over degradation (2, 3). The driving factors leading to pathological fibrosis are object of controversies (4, 5), but it is likely that inflammatory mediators including, but not exclusively, interleukin-17A (IL-17A) (6), transforming growth factor-β 1 (TGF-β) (7) and IL-6 (8, 9) play a major role (10).

IL-17A is a pro-inflammatory cytokine mainly but not exclusively produced by Th17 cells involved in protection against extracellular bacteria and fungi as well as in autoimmunity (11). IL-17A levels and/or Th17 cells have been reported to be increased in SSc peripheral blood, bronchoalveolar lavage fluid, and skin (12–18), although some reports point to decreased serum levels (19, 20).

The role of IL-17A in the development of fibrosis is controversial (21). Concordant data generated in various animal models of fibrosis point to a pro-fibrotic activity (22–24). In contrast, studies in humans using *in vitro* fibroblast cultures suggest that IL-17A rather controls fibrosis (24–26) by inhibiting collagen synthesis and the transdifferentiation of fibroblasts to myofibroblasts induced by TGF-β (18, 24, 26). Furthermore, the number of IL-17A+ cells appears to be inversely correlated to the extent of skin fibrosis (18) and to increase with disease duration (27), thus pointing to an antifibrotic activity *in vivo*. Nonetheless, IL-17A and Th17 cells have potent pro-inflammatory properties including the induction of several mediators dysregulated in SSc, including IL-8, IL-6, monocyte chemotactic protein-1 (MCP-1), matrix metalloproteinases (MMP) by dermal fibroblasts, in addition to enhancing their proliferation capacity (12, 26, 27).

TGF-β is considered a master pro-fibrotic cytokine with important immunomodulatory properties regulating inflammation, adipogenesis, chondrogenesis, osteogenesis, epithelial cell differentiation and proliferation, hemopoiesis, and wound healing. It binds to TGF-β receptor type-2 (TGFβR2), thus recruiting and phosphorylating signal transducer TGF-β receptor type-1 (TGFβR1). TGF-β is secreted as a latent protein, which needs to be activated mostly by protease-mediated cleavage favored by integrin-mediated release (28). It was reported that the levels of TGF-β are increased in the skin of SSc patients (7, 29–32) and that TGF-β-induced gene signature is strongly increased in SSc skin and positively correlates with the severity of the disease (33). A recent pilot trial targeting TGF-β with fresolimumab has shown some efficacy in reducing skin fibrosis (34).

IL-6 is a multifunction cytokine that plays a key role in acute phase responses, regulates cell proliferation, activation, and differentiation (35), and IL-6 serum levels are increased in SSc (8, 9, 36). Furthermore, IL-6 induces collagen production by dermal fibroblasts in the presence of trans-signaling by soluble IL-6 receptor (37) and participates to imbalanced degradation of ECM that is controlled by MMP and their inhibitors (38). IL-6 blockade by both passive or active immunization strategies or IL-6 genetic deletion reduces fibrotic responses in animal models of fibrosis (39–41). Furthermore, an IL-6 targeted therapy in SSc appears to be promising (42) and is currently assessed in a phase 3 clinical trial (NCT02453256).

The interplay between IL-17A and TGF-β has been only partially assessed (18, 24) and here we address the question to which extent their coordinate action affects fibroblast responses. We report for the first time that they may simultaneously have synergic or antagonistic activities depending on the readout used to evaluate fibroblast responses.

## Materials and Methods

### Patients

Nine SSc individuals and nine healthy controls were included in this study. All patients met the ACR/EULAR classification criteria for SSc (43) and their clinical presentation classified according to the criteria proposed by LeRoy et al. (44). Clinical characteristics of the patients are shown in Table 1. A biopsy was performed in the affected skin of SSc individuals. The control group consisted of age and sex matched individuals who underwent corrective abdomen or breast surgery at the department of plastic surgery of HUG in Geneva (Switzerland). None of the healthy individuals had dermatological disorders and none was under immunosuppressive agents or glucocorticoids. This study was approved by the ethical committee of the institutions involved (06-063, Commission cantonale d’éthique de la recherche, Geneva, Switzerland) and was conducted according to the Declaration of Helsinki. Written informed consent was obtained from each individual.

**Table 1:** Clinical characteristic of the fibroblast donors.

	SSc	HD
Age, mean (range), years	61.2 (46–78)	42.1 (26–60)
Sex (M/F)	3/6	1/8
Disease duration, mean (range), months	108 (12–324)	N/A
Form (limited/diffuse)	4/5	N/A
MRSS (mean, range)	12.4 (4–28)	N/A
ANA positivity (yes/no) (*n* = 8)	7/1	N/A
ANA specificity (ACA/ATA) (*n* = 6)	4/2	N/A
DLCO, mean (range), % of reference (*n* = 8)	70.8 (33–112)	N/A
Synovitis	3 of 8	N/A
CK elevation	1 of 9	N/A
DU	3 of 9	N/A
ILD	2 of 8	N/A
GERD	6 of 8	N/A
Prednisone use	4 of 9	none
Prednisone dose >10 mg/day	0 of 9	none
Previous use of immunosuppressive agents	4 of 9	none

*DU and synovitis were clinically defined, disease duration refers to the time from the onset of the first non-Raynaud’s disease manifestation, ILD was assessed by high resolution CT scan, GERD was determined by gastroscopy. ACA, anticentromere antibody; ANA, antinuclear antibodies; ATA, antitopoisomerase antibody; CK, creatine kinase; DLCO, diffusing capacity of the lungs for carbon monoxide; DU, digital ulcer; GERD, gastroesophageal reflux disease; ILD, interstitial lung disease; IS, immunosuppressant agents; MRSS, modified Rodnan skin score; N/A, not available; HD, healthy donors; SSc, systemic sclerosis*.

### Reagents

rhIL-17A, rhTGF-β, monoclonal mouse IgG1 TGF-β1, 2, 3 antibody, IL-6, MCP-1, MMP-1, IL-8, pro-collagen Iα1 and fibronectin ELISA DuoSet kits were from R&D Systems (Abingdon, UK); DMEM, PBS, glutamine, penicillin, streptomycin, trypsin, dispase, collagenase type I from Gibco (Paisley, UK); FCS from Biowest (Nuaillé, France); BSA, α-ketoglutaric acid, β-amino propionitrile, l-ascorbic acid, p38 MAPK inhibitor SB203580, and PI3K inhibitor LY294002 from Sigma (St. Louis, MO, USA); MEK1/2 inhibitor U-0126 from Calbiochem (San Diego, CA, USA); TGF-βRI inhibitor SD 208, JNK inhibitor SP 600125 and IKK-2 inhibitor TPCA-1 from Tocris Bioscience (Bristol, UK); LEAF irrelevant control mAbs from Biolegend (San Diego, CA, USA); Complete Protease Inhibitor Cocktail and PhosSTOP phosphatase inhibitor from Roche (Basel, Switzerland); nitrocellulose membranes and chemiluminescence (ECL) blotting analysis system from GE Healthcare (Zurich, Switzerland); phospho-Akt (Ser473), phospho-Smad2 (Ser465/467), phospho-p38 MAPK (Thr180/Tyr182), phospho-NF-κB p65 (Ser536), phospho-IκB-α (Ser32), β-actin and BSA for Western blots from Cell Signaling (Danvers, MA, USA); TMB ELISA substrate from Abcam (Cambridge, UK); EZ4U cell proliferation assay from Biomedica (Vienna, Austria).

### Cell Cultures

Human fibroblasts were isolated from skin, as previously described (26). Cells were cultured in DMEM containing 10% FCS, 1% non-essential amino acids, 1% l-glutamine, 1% sodium pyruvate, 50 U/ml penicillin, and 50 µg/ml streptomycin. Fibroblasts were used at passage 5–8. Twenty thousand cells/well were seeded in 96-well plates for 24 h, then starved for 16 h in the absence of FCS, followed by stimulation with IL-17A (25 ng/ml) and/or TGF-β (2.5 ng/ml). EZ4U cell proliferation assay was used to determine the viability of fibroblasts. When used, inhibitors (SD208, U0126, SB203580, SP600125, Ly294002, TPCA-1) at indicated doses, vehicle (DMSO), 10 µg/ml of TGF-β 1 neutralizing antibody or 10 µg/ml of an irrelevant control antibody were added for 1 h prior to stimulation with IL-17A (25 ng/ml) or TGF-β (2.5 ng/ml), in triplicates. Culture supernatants were harvested after 48 h.

### ELISA and Western Blot

IL-6, MCP-1, IL-8, MMP-1, pro-collagen 1αI, and fibronectin were quantified using DuoSet ELISA kits, according to the manufacturer instruction (R&D Systems, Abingdon, UK). For Western blot, cell cultures were treated as previously described (26). Briefly, 20 µg of total protein extract were separated in 10% reducing SDS-PAGE and electroblotted onto nitrocellulose membranes. Blots were incubated with antibodies against phospho-Akt (Ser473), phospho-Smad2 (Ser465/467), phospho-p38 MAPK (Thr180/Tyr182), phospho-NF-κB p65 (Ser536), phospho-IκB-α (Ser32), and β-actin. Horseradish peroxidase-conjugated mouse or rabbit antisera were used to reveal primary binding, followed by detection by an ECL blotting analysis system. Quantification analysis was performed with ImageJ software (http://rsbweb.nih.gov/ij), and values were normalized to β-actin.

### Statistical Analysis

Statistical analysis was performed with GraphPad Prism version 7.02 (Graphpad Software, La Jolla, CA, USA). Shapiro–Wilk normality test was used to evaluate if the residuals follow a Gaussian distribution. Statistical significance was assessed by paired Student’s *t*-test. *P* values <0.05 were considered statistically significant.

## Results

### IL-6 and MCP-1 Are Synergistically and Specifically Induced in Human Fibroblasts by the Combined Action of IL-17A and TGF-β

**Figure 1:**
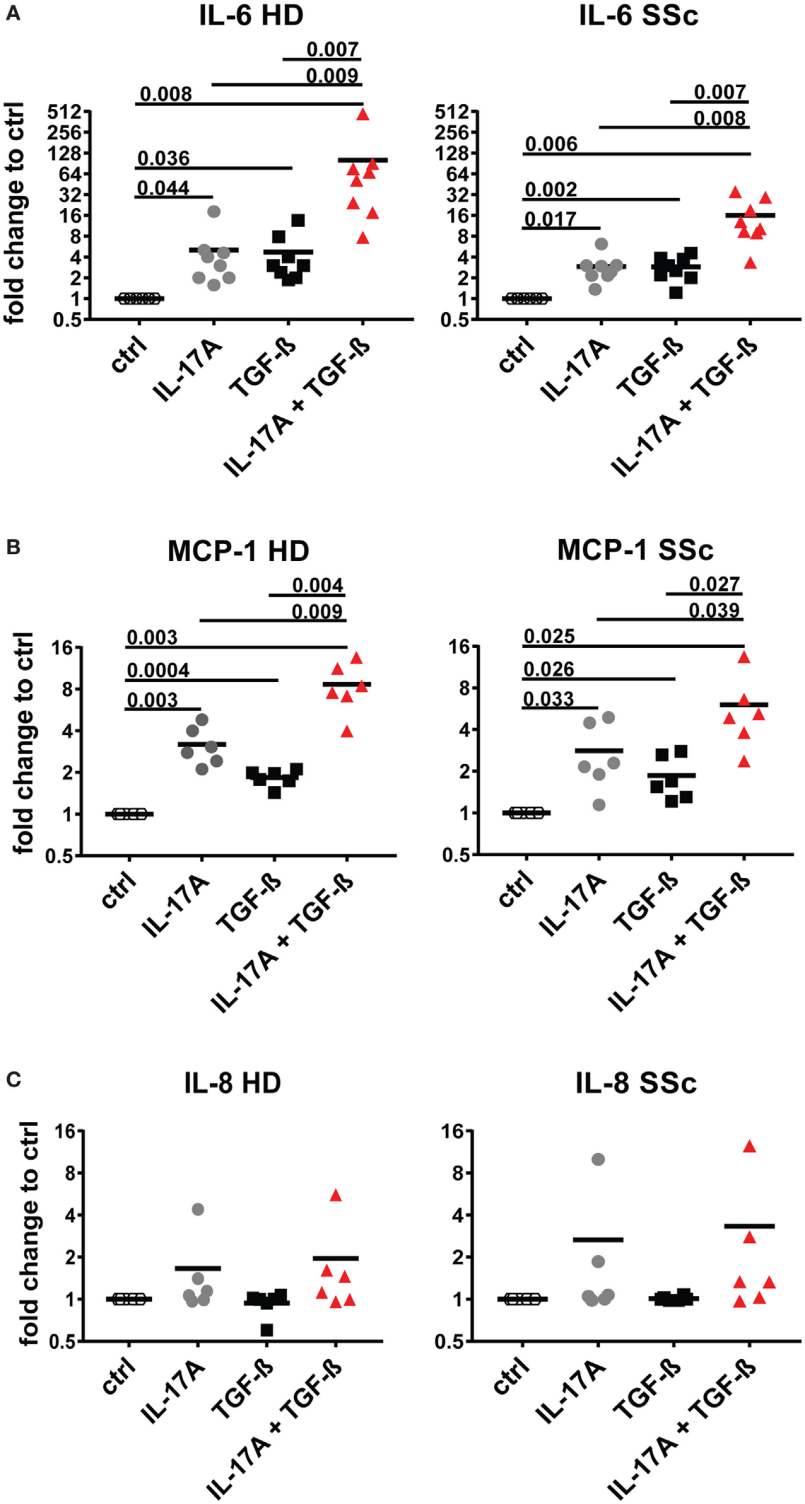
IL-6 and monocyte chemotactic protein-1 (MCP-1) are synergistically and specifically induced in human fibroblasts by the combined action of IL-17A and TGF-β. Primary human dermal fibroblasts from healthy donors (HD) and systemic sclerosis (SSc) patients were cultured in the presence of IL-17A (25 ng/ml), TGF-β (2.5 ng/ml), or their combination for 48 h, in 96-well plates, in triplicates. IL-6 **(A)**, MCP-1 **(B)**, IL-8 **(C)** were assessed by ELISA in culture supernatants. Results are expressed as fold change compared to spontaneous production in control (ctrl) cultures. Basal levels were: 22.7 (±7.3) and 40.7 (±16.7) pg/ml for IL-6; 328.1 (±33.3) and 377.2 (±85.4) pg/ml for MCP-1 and 211.8 (±56.6) and 207.7 (±56.9) pg/ml for IL-8, in HD and SSc, respectively. Significance was assessed by paired *t* test.

**Figure 2:**
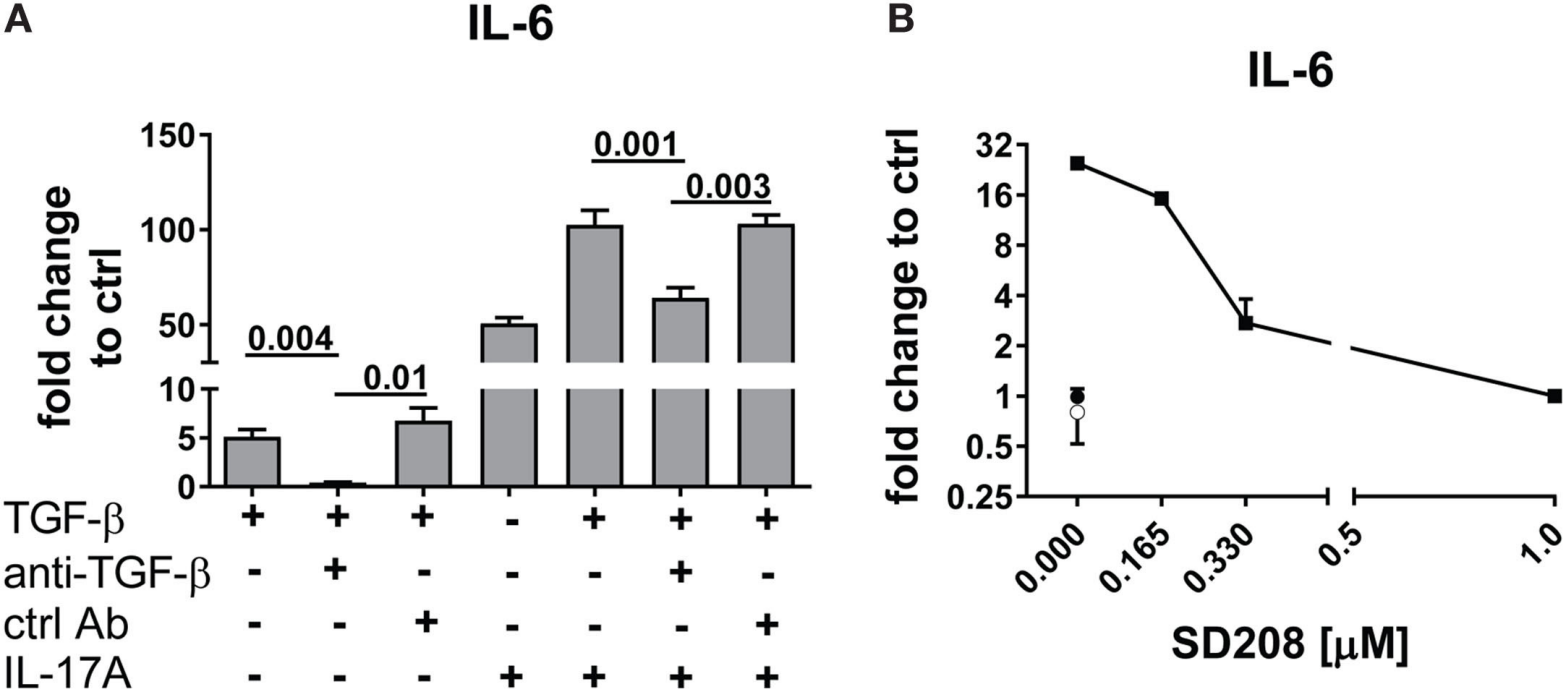
TGF-β inhibition abrogates the synergistic response with IL-17A. HD fibroblasts were treated with **(A)** 10 µg/ml TGF-β 1 neutralizing antibody or 10 µg/ml of an irrelevant ctrl Ab, **(B)** SD208 (TGFβR1 inhibitor) or vehicle for 1 h prior to the addition of IL-17A (25 ng/ml) or TGF-β (2.5 ng/ml) and cultured for 48 h. IL-6 levels in SN were assessed by ELISA. Results are shown as fold change to untreated control cultures (basal level of IL-6 was 3.1 ± 1.1 pg/ml). **(B)** Square: TGF-β (10 ng/ml); empty circle: (SD2018, 1 µM); full circle: vehicle. Significant differences were assessed by paired *t*-test. Bars in **(A)** and symbols in **(B)** represent the mean + SEM of three experiments.

Interleukin-17A and TGF-β are both considered of pathogenic importance in SSc. However, relatively little is known on their effects when applied jointly to fibroblasts. To address this issue, we used the fibroblast production of IL-6, MCP-1, and IL-8 as read out and we observed that the joint presence of suboptimal doses of IL-17A and TGF-β induced synergic responses specifically for IL-6 and MCP-1, but not for IL-8 (Figure 1). Suboptimal doses of agonistic cytokines were chosen in order to avoid maximal fibroblast responses—as observed with higher levels of IL-17A (data not shown)—thus potentially favoring the quantification of synergistic or antagonistic activities. Of interest, although SSc fibroblasts produced higher basal levels of IL-6, both SSc and HD cells responded equally well to the combination. In particular, it has to be noted that IL-17A and TGF-β were equipotent when used separately to induce the production of IL-6 and their joint action consistently over-enhanced the production of IL-6 by up to 32-folds (Figure 1A). To prove that the responses to TGF-β were not due to contaminants, we performed two distinct assays. First, TGF-β neutralization by a specific antiserum abrogated the production of IL-6 induced by TGF-β used alone and reduced the production of IL-6 to the levels induced by IL-17A when used in combination with IL-17A (Figure 2A). Second, inhibition of TGFβR1 signaling by SD208 reduced IL-6 levels in a dose-dependent manner (Figure 2B). All together, these results indicate that TGF-β acts *via* its specific receptor and, most notably, that TGF-β synergizes with IL-17A to induce the fibroblast production of IL-6.

### Common and Private Signaling Pathways Are Used by IL-17A and TGF-β to Induce IL-6

**Figure 3:**
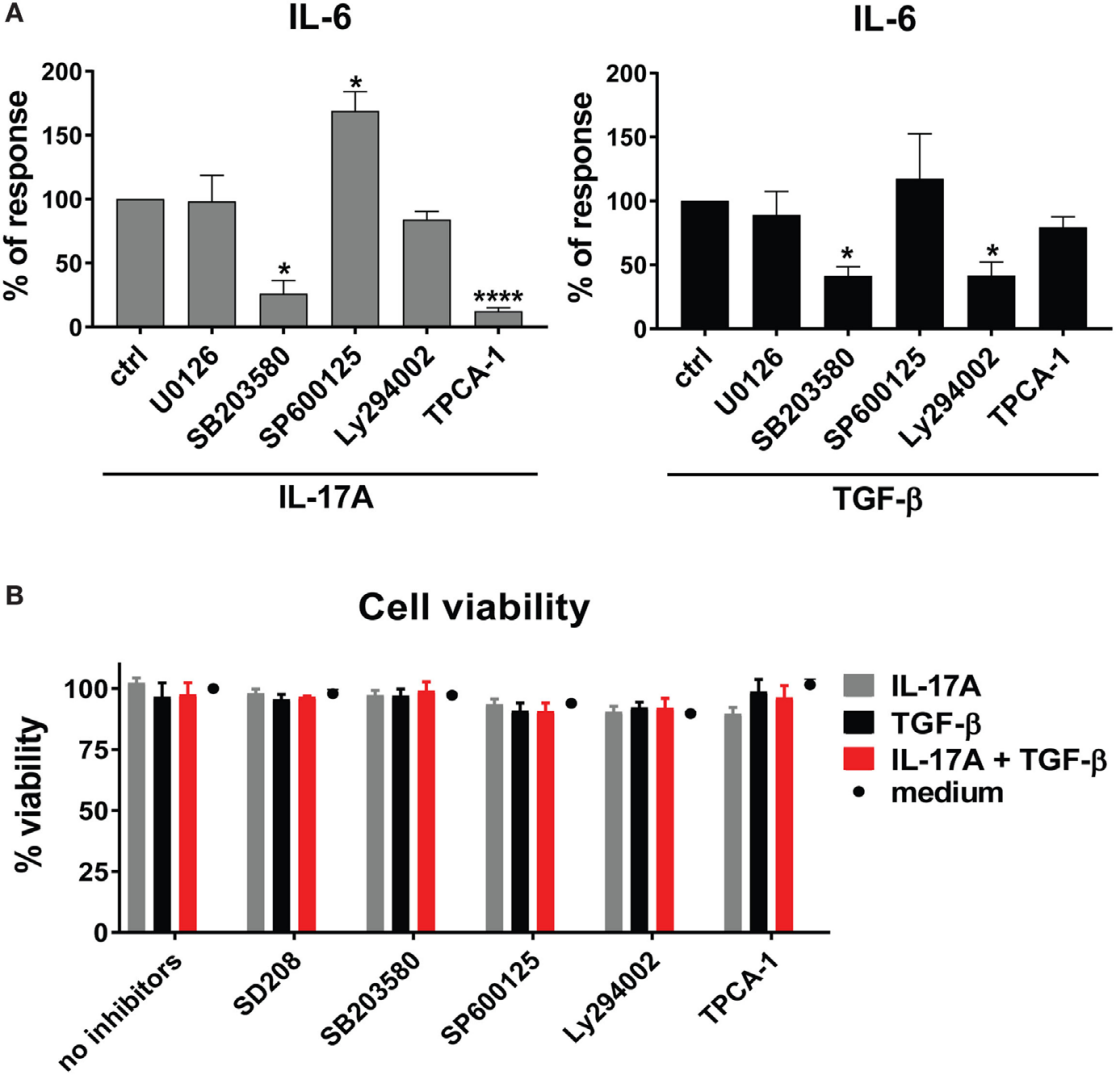
Shared and private signaling pathways are preferentially used by IL-17A and TGF-β to induce IL-6. Healthy donors fibroblasts were treated with optimal doses of inhibitors (20 µM U0126, 20 µM SB203580, 10 µM SP600125, 10 µM Ly294002, or 0.37 µM TPCA-1) or vehicle for 1 h prior to the addition of IL-17A (25 ng/ml) or TGF-β (2.5 ng/ml) and cultured for an additional 48 h, in triplicates. **(A)** IL-6 levels in SN were assessed by ELISA. Results are shown as the percentage of IL-6 production induced by IL-17A or TGF-β in the absence of inhibitors (levels of IL-6 were: 22.8 ± 3.3 pg/ml for IL-17A and 8.8 ± 3.9 pg/ml for TGF-β). Bars represent the mean + SEM of three experiments. Significant differences versus control were assessed by paired *t*-test: *P < 0.05, ****P < 0.001. **(B)**. Fibroblast viability was assessed by EZ4U and found >90% for all culture conditions.

To unravel the mechanisms explaining the actions of IL-17A and TGF-β, we tested whether signaling pathways known to be involved in IL-6 transcription were used by IL-17A and TGF-β. We focused on IL-6 since it is considered a promising candidate target in SSc treatment (42). We found that, in HD fibroblasts, inhibition of NFκB by TPCA-1 preferentially inhibited the response to IL-17A (Figure 3A, left panel), while inhibition of PI3K/Akt by Ly294002 preferentially inhibited the response to TGF-β (Figure 3A, right panel). Interestingly, inhibition of p38 MAPK by SB203580, reduced the responses to both IL-17A and TGF-β (Figure 3A). By contrast, the inhibition of MEK1/2 with U0126 did not influence the production of IL-6, while inhibition of JNK by SP600125 increased its production in response to IL-17A, but not to TGF-β (Figure 3A). Importantly, fibroblast viability was found >90% for all culture conditions (Figure 3B).

### NFκB and PI3K/Akt Signaling Pathways Are Privately Used by IL-17A and TGF-β and Synergize in the Induction of IL-6

**Figure 4:**
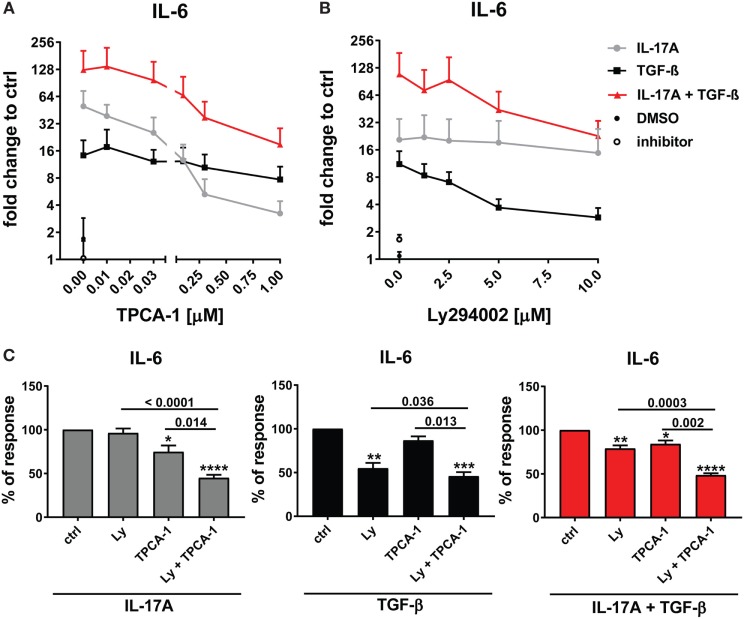
NFκB and PI3K signaling pathways are preferentially used by IL-17A and TGF-β, respectively and together cooperate in inducing IL-6. Healthy donors fibroblasts were treated with the indicated concentrations of **(A)** TPCA-1; **(B)** Ly294002; or **(C)** suboptimal doses of TPCA-1 (0.03 µM) and/or Ly294002 (Ly, 2 µM) for 1 h prior to addition of IL-17A (25 ng/ml) and/or TGF-β (2.5 ng/ml). After 48 h, culture SNs were collected and IL-6 levels were assessed by ELISA. **(A,B)** Results are shown as fold change to untreated cells, mean + SEM is indicated (*N* = 4). Please note the log_2_ scale. **(C)** Results are shown as the percentage of IL-6 production induced by IL-17A and/or TGF-β in the absence of inhibitors (levels of IL-6 were: 86.2 ± 11.8 pg/ml for IL-17A, 49.6 ± 10.2 pg/ml for TGF-β, and 257.5 ± 82.5 pg/ml for IL-17A + TGF-β). Bars represent the mean + SEM. Significant differences versus control were assessed by paired *t*-test: *P < 0.05, **P < 0.01, ***P < 0.001, ****P < 0.001.

The preferential use of NFκB by IL-17A and PI3K/Akt by TGF-β to stimulate the production of IL-6 (Figure 3A) prompted us to test whether these signaling pathways were involved in the synergistic action of IL-17A and TGF-β. The inhibition of NFκB (Figure 4A) and PI3K/Akt in HD fibroblasts (Figure 4B) decreased in a dose-dependent manner the production of IL-6 driven by the combined action of IL-17A and TGF-β. Notably, in these experiments, PI3K/Akt inhibition by itself did not affect the response to IL-17A, but when combined with NFκB inhibition, it further decreased the production of IL-6 (Figure 4C, left panel). Reciprocally, NFκB inhibition by itself did not affect the response to TGF-β, but when combined with PI3K/Akt inhibition, it further decreased the production of IL-6 (Figure 4C, middle panel). Moreover, the combined inhibition of NFκB and PI3K/Akt substantially reduced the production of IL-6 triggered by the joint presence of IL-17A and TGF-β (Figure 4C, right panel). Of note, suboptimal doses of inhibitors were used to perform the above experiments to favor the assessment of additive outcomes and decrease the likelihood of off-target effects. Furthermore, to ensure the specificity of the inhibitors used, we performed phospho-blot analysis. We observed that Ly294002 specifically inhibited the phosphorylation of Akt, while TPCA-1 specifically inhibited the phosphorylation of IκBα and the downstream p65 transcription factor of the NFκB complex (Figure 6A). Thus, NFκB and PI3K/Akt signaling converge in inducing the production of IL-6 by HD fibroblasts in the presence of IL-17A and TGF-β.

### p38 MAPK Signaling Pathway Is Common to IL-17A- and TGF-β-Induced IL-6 Production

**Figure 5:**
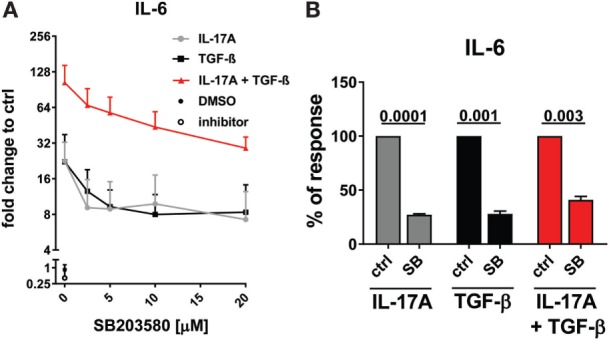
p38 MAPK signaling pathway is common to IL-17A- and TGF-β-induced IL-6 production. Healthy donors fibroblasts were treated with the indicated concentrations of SB203580 **(A)** or 20 µM SB203580 **(B)** for 1 h prior to the addition of IL-17A (25 ng/ml) and/or TGF-β (2.5 ng/ml) in triplicates. After 48 h, culture SNs were collected and IL-6 levels were assessed by ELISA. **(A)** Results are shown as fold change to untreated cells, mean + SEM is indicated, *N* = 3. Please note the log_2_ scale. **(B)** Results are shown as the percentage of IL-6 production induced by IL-17A and/or TGF- β in the absence of inhibitor (levels of IL-6 were: 14.7 ± 7.4 pg/ml for IL-17A, 11.4 ± 6.1 pg/ml for TGF-β, and 48.9 ± 13.7 pg/ml for IL-17A + TGF-β). Bars represent the mean + SEM of three experiments. Significant differences versus control were assessed by paired *t*-test.

**Figure 6:**
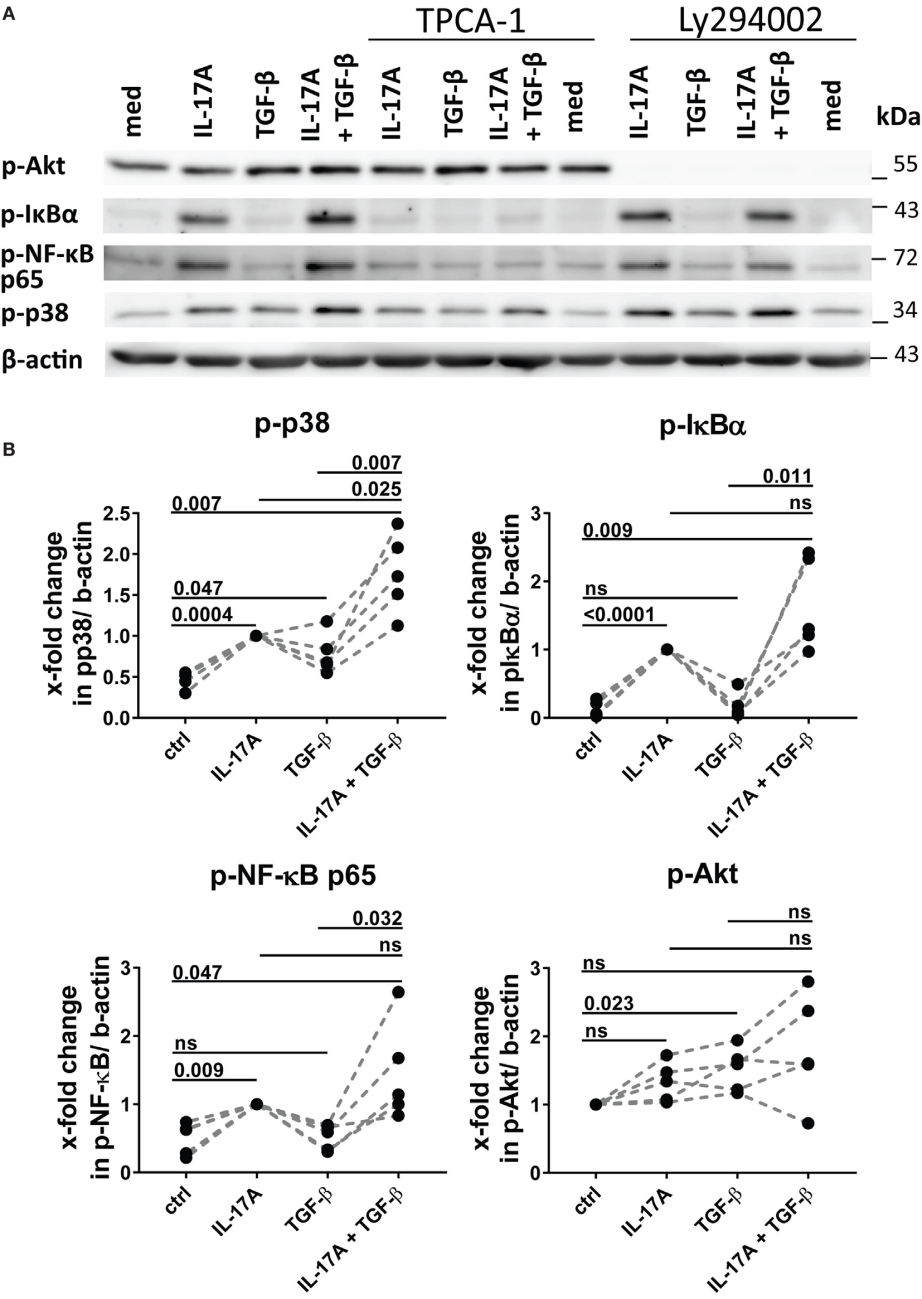
Phosphorylation of MAPK p38 is enhanced by the combined action of IL-17A and TGF-β. **(A)** Western blot (WB) of healthy donors fibroblasts treated with 1 µM TPCA-1 and/or 10 µM Ly294002 for 1 h prior to addition of IL-17A (25 ng/ml) and/or TGF-β (2.5 ng/ml) and cultured for an additional 10 min. Results are representative of three experiments with inhibitors and two additional experiments with cytokines only. **(B)** Quantification of Western blot (WB) analysis was performed with ImageJ software (http://rsbweb.nih.gov/ij) and values were normalized to β-actin, *N* = 5. Results are shown as fold change to IL-17A-treated cells (for p-p38, p-IκBα, and p-NF-κB p65) or to untreated cells (for p-Akt), *N* = 5. Significance assessed by paired *t* test.

As reported above, p38 MAPK inhibition reduced the production of IL-6 induced by both IL-17A and TGF-β (Figure 3A). Not unexpectedly, substantial inhibition of IL-6 production was observed and this effect was dose-dependent, when HD fibroblasts were stimulated jointly by IL-17A and TGF-β (Figures 5A,B). Consistently with the inhibition experiments, the combined stimulation of HD fibroblasts by IL-17A and TGF-β resulted in 1.8-fold increase (*P* = 0.025) of p38 MAPK phosphorylation, when compared to levels of phosphorylated p38 MAPK induced by IL-17A alone, as assessed by Western blot (Figure 6). Furthermore, the enhanced phosphorylation of p38 MAPK in the joint presence of IL-17A and TGF-β was unique among the signaling molecules we examined (Figure 6). Thus, p38 MAPK is used by both IL-17A and TGF-β, separately and jointly, to induce the production of IL-6 by HD fibroblasts.

### IL-17A Decreases SMAD2 Phosphorylation and Production of Type-I Collagen and Fibronectin Triggered by TGF-β

**Figure 7:**
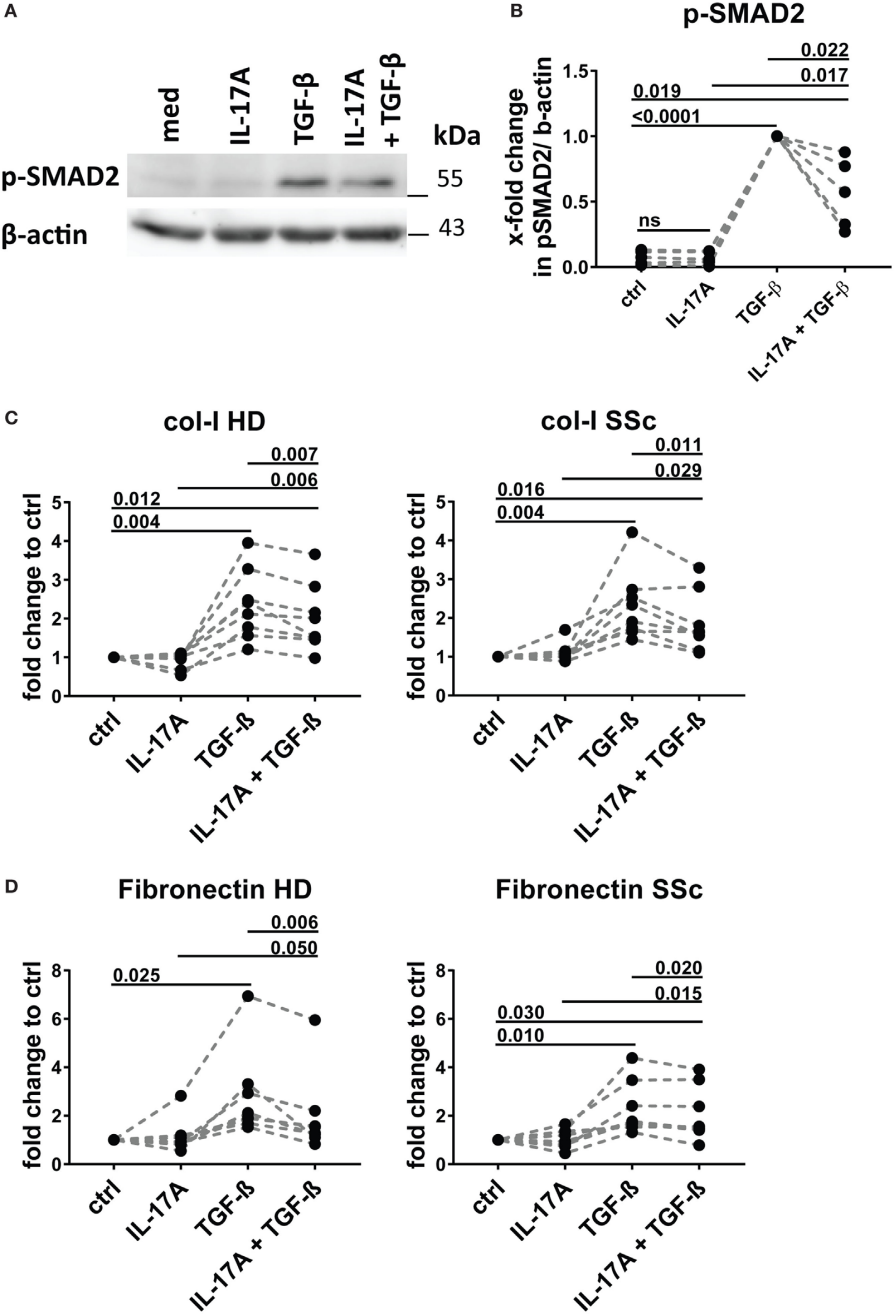
IL-17A inhibits SMAD2 phosphorylation and production of type I collagen and fibronectin induced by TGF-β. Healthy donors (HD) fibroblasts were treated with IL-17A (25 ng/ml) and/or TGF-β (2.5 ng/ml) for 10 min. **(A)**. Western blot (WB) representative of five distinct experiments. **(B)** Quantification of WB analysis. Results are shown as fold change to TGF-β-treated cells, *N* = 5. **(C,D)** Primary human fibroblasts from HD (left panel) and systemic sclerosis (SSc) patients (right panel) in triplicates were treated with IL-17A (25 ng/ml), TGF-β (2.5 ng/ml), or a combination of these cytokines. After 48 h, culture supernatants were collected and type I collagen **(C)** and fibronectin **(D)** levels were assessed by ELISA (*N* = 5). Results are shown as fold change to untreated cells. Basal levels for col-I were 364.8 (±91.0) and 203.8 (±66.5) ng/ml and for fibronectin 972.7 (±273.9) and 1036,4 (±307.6) ng/ml, in HD and SSc, respectively. Significance was assessed by paired *t*-test.

**Figure 8:**
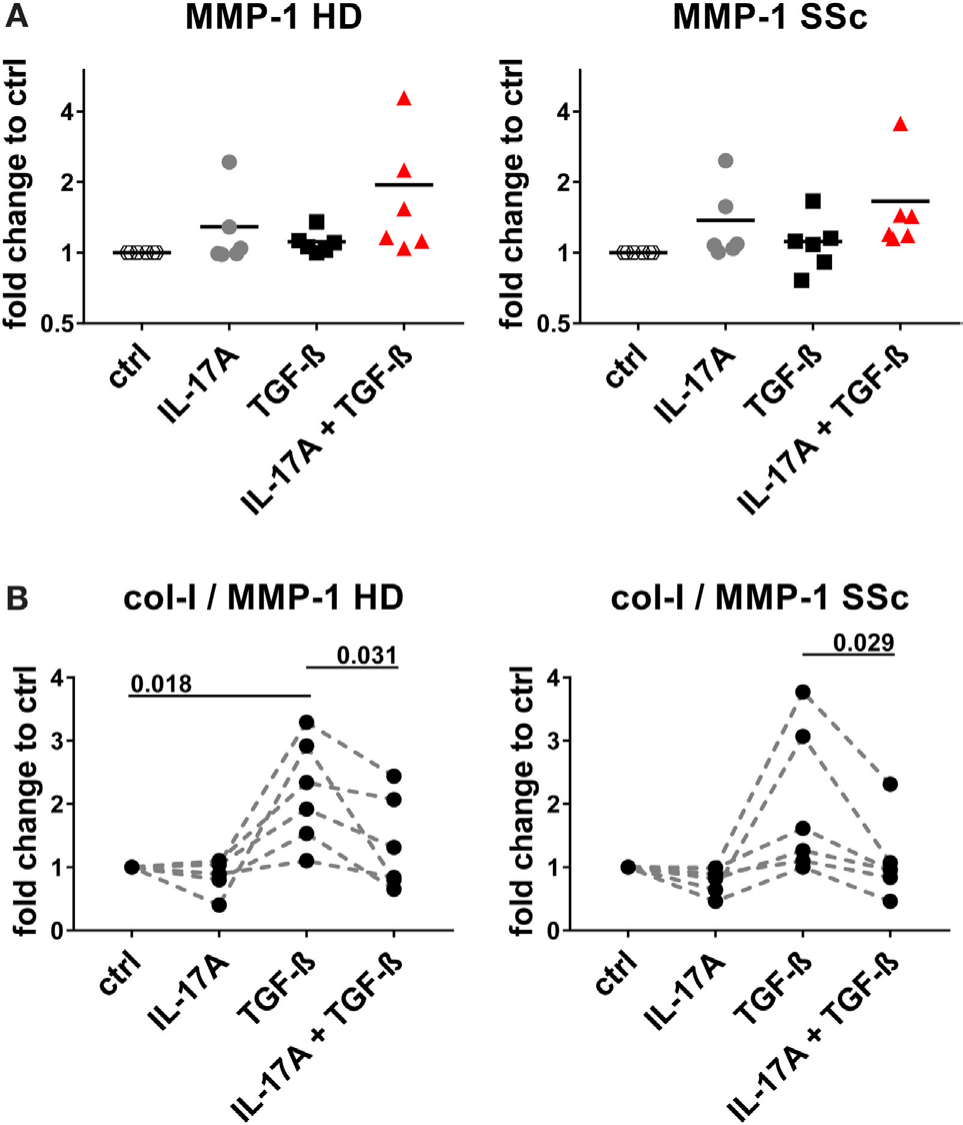
IL-17A decreases the col-I to MMP-1 ratio enhanced by TGF-β. Primary human dermal fibroblasts from healthy donors (HD) (left panel) and systemic sclerosis (SSc) patients (right panel) were cultured in the presence of IL-17A (25 ng/ml), TGF-β (2.5 ng/ml), or their combination for 48 h, in 96-well plates, in triplicates. MMP-1 levels **(A)** were assessed by ELISA in culture supernatants. Results are expressed as fold change compared to spontaneous production in control (ctrl) cultures. Basal levels for MMP-1 were 15.71 (±1.3) and 20.1 (±2.3) ng/ml, in HD and SSc, respectively. **(B)** The ratio of col-I levels from Figure 7C to MMP-1 was calculated. Significance was assessed by paired *t*-test.

We and others have reported that IL-17A may decrease the fibroblast response to TGF-β when the ECM response is taken into consideration. We, therefore, explored in the same experimental settings in which IL-17A and TGF-β were displaying synergic activities for the induction of IL-6, whether the same hold true concerning collagen production. The canonical signaling pathway of TGF-β leads to SMAD2 phosphorylation and type-I collagen (col-I) production. We observed that in HD fibroblasts cultured in the joint presence of TGF-β and IL-17A, the phosphorylation of SMAD2 decreased by 0.6-fold (*P* = 0.022) when compared to that induced the TGF-β alone (Figures 7A,B). In addition, extending previous reports, IL-17A decreased significantly the production of col-I robustly induced by TGF-β. Most importantly, this was observed in both, HD (*P* = 0.007) and SSc (*P* = 0.011) fibroblasts (Figure 7C). Very interestingly, fibronectin was regulated by IL-17A and TGF-β in a similar manner as col-I. IL-17A did not modify fibronectin production, while it decreased the exuberant response induced by TGF-β (Figure 7D). Furthermore, when we looked at the ratio of col-I to MMP-1, as a surrogate of ECM turnover, we observed that this ratio was increased in the presence of TGF-β and significantly decreased when IL-17A was added to TGF-β (Figures 8A,B). Thus, our data support a dual role in the relationship between IL-17A and TGF-β. On the one hand, they cooperate in inducing IL-6, on the other hand, they exert opposite functions in inducing type-I collagen and fibronectin.

## Discussion

**Figure 9:**
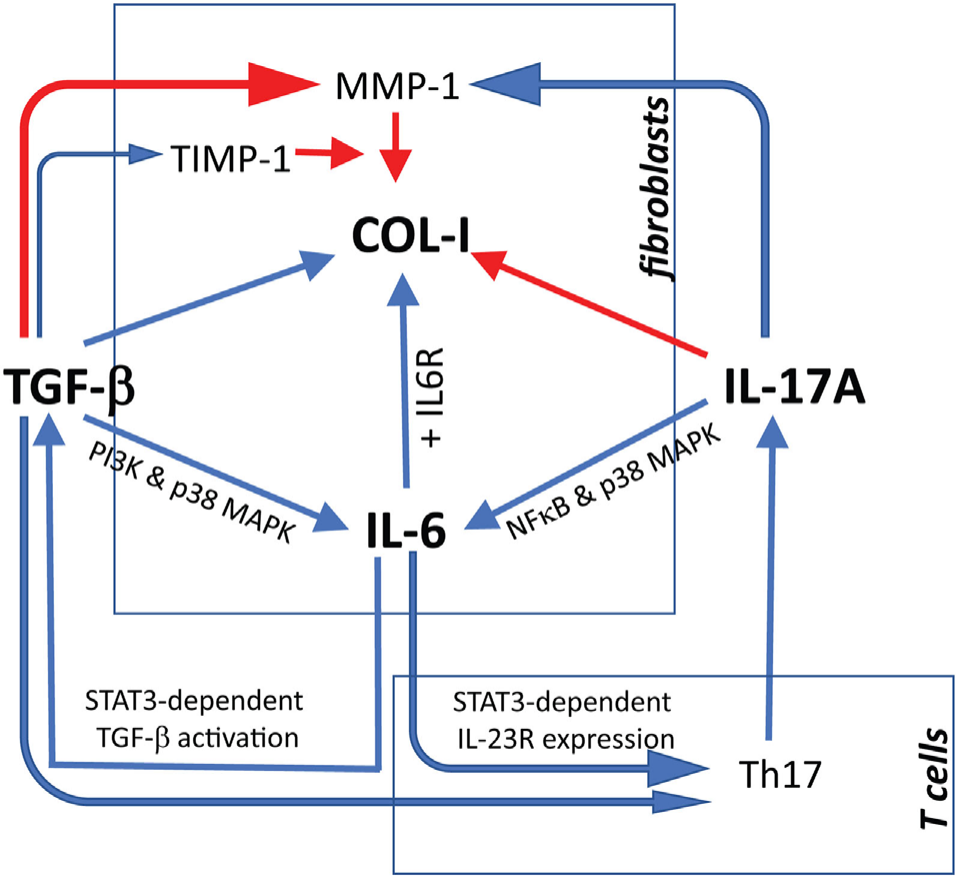
Proposed model linking IL-17A, TGF-β, and IL-6 in the context of extracellular matrix deposition and Th17 cell differentiation. Blue arrows: stimulatory signal; red arrows: inhibitory signal. The relevant references are reported in the discussion. For tissue inhibitor of metalloproteinases 1 (TIMP-1), we refer to Fineschi et al. (45).

We show here for the first time a synergistic activity of IL-17A and TGF-β for the production of IL-6 (and MCP-1) by SSc and HD dermal fibroblasts, an effect, at least in part, dependent on the convergent signaling mediated by p38 MAPK, NFκB, and PI3K/Akt, as examined in HD. Additionally, our data show an inhibitory role of IL-17A in fibrosis, particularly for its negative effect on TGF-β-mediated col-I production dependent on SMAD signaling (Figure 9).

IL-17A is well known to stimulate IL-6 production by synovial-like fibroblasts (46) as well as HD and SSc dermal fibroblasts (27), in addition to other cell types (11). However, relatively few studies essentially performed with lung fibroblasts have documented the capacity of TGF-β to induce IL-6 production (47, 48). Our data are in agreement with such studies and prove that IL-6 production induced by TGF-β is dependent on TGFβR1 and mediated, at least in part, by PI3K/Akt and p38 MAPK-signaling pathways. Interestingly, in one previous study, TGF-β exerted a dual role, favoring IL-6 production when used alone, but reducing the production of IL-6 in fibroblast vigorously stimulated by IL-1 (47). We have not extensively explored the capacity of TGF-β to modulate the production of IL-6 induced by optimally activated fibroblasts by other cytokines, but we robustly observed a synergistic effect with IL-17A within the dose used.

The synergy of IL-17A and TGF-β on IL-6 production is reported for the first time in this study and observed in both, HD and SSc fibroblasts. While we show the preferential use of NFκB by IL-17A and PI3K/Akt by TGF-β to stimulate the production of IL-6, the combined inhibition of NFκB and PI3K/Akt substantially reduced the production of IL-6 triggered by the joint presence of IL-17A and TGF-β. Interestingly, we report that p38 MAPK signaling pathway was common for IL-17A-, TGF-β-, and combined IL-17A/TGF-β-induced IL-6 production and the phosphorylation of p38 MAPK was significantly higher in the joint presence of IL-17A and TGF-β, when compared to IL-17A alone. Thus, we propose a model where IL-17A synergizes with TGF-β to produce IL-6 by dermal fibroblasts using the common p38 MAPK transduction pathway in addition to the preferentially private use of NFκB and PI3K/Akt by IL-17A and TGF-β, respectively. Of note, inhibition of JNK by SP600125 resulted in higher levels of IL-6, thus suggesting that the expression of IL-6 depends on the fine balance of positive and negative regulators. The signaling studies were performed with HD fibroblasts. However, the convergence of these pathways may be at work also in SSc contributing to higher levels of IL-6.

The canonical TGF-β signaling pathway relies on phosphorylation of SMAD family proteins, thus contributing to col-I production (49–51). Our data show that IL-17A decreases TGF-β induced phosphorylation of SMAD2. Consistently with lower SMAD2 phosphorylation levels, both SSc and HD fibroblasts produced reduced levels of col-I and fibronectin in the presence of IL-17A and TGF-β, when compared to TGF-β alone. The mechanisms associated with SMAD2 reduced phosphorylation induced by IL-17A deserve further investigations. However, the inhibitory effect of IL-17A on TGF-β-triggered col-I production supports the data published by Truchetet et al. where IL-17A was shown to decrease TGF-β-induced α-SMA transcriptional activity in fibroblasts (18) and extend the reported IL-17-dependent inhibition of spontaneous col-I production by HD dermal fibroblasts reported by Nakashima et al. (24). However, at variance with Nakashima data, we observed the inhibitory effect of IL-17A on both HD as well as SSc fibroblast. In this respect is important to note that in our experiments the spontaneous col-I production was lower in SSc compared to HD fibroblasts.

Altogether, concerning fibrotic responses, the bulk of data generated *in vitro* with human material indicate that IL-17A has two facets. On the one hand, by itself, it increases inflammation inducing several pro-inflammatory cytokines and MMPs. In addition, it synergizes with TGF-β, as we show here, to further increase the production of IL-6 and MCP-1, which roles in fibrosis have been extensively reviewed (35, 52). On the other hand, it has direct inhibitory effects on TGF-β-triggered col-I production. Furthermore, IL-6 may enhance the activation of TGF-β in a STAT3-dependent mechanisms (53), and enhance Th17 cell proliferation by increasing IL-23 receptor expression on T cells (54). Additionally, TGF-β may play also a role in Th17 differentiation program by enhancing RORγT expression (55). Thus, IL-6 and TGF-β may cooperate to polarize and expand Th17 cells. In turn, IL-17A produced by Th17 cells may simultaneously exert antifibrotic responses by inhibiting collagen and pro-fibrotic responses by enhancing IL-6 production (Figure 9). An obvious limitation to this scenario is the minimalistic *in vitro* approach used to generate our data in which fibroblast monolayers are submitted to the influence of a limited number of effector molecules. To overcome such limitation, more complex experimental systems have to be adopted in which fibroblasts should be submitted to stimulation in three dimensional cultures and under the influence of other cell types, which likely play a role in modulating ECM production. This is of outmost importance, since substantial discrepancies have been highlighted when human and *in vivo* murine models are compared for the role of IL-17A in fibrosis (21). It may be argued that no differences were observed in our settings when SSc and HD responses were compared. However, we think that our observations are relevant to SSc since we and other have documented increased levels of the agonist used, namely TGF-β and IL-17A in SSc tissues compared to HD. Differences in the amount of these cytokines may result in stronger responses *in vivo*. The limited number of SSc samples (4 lSSc and 5 dSSc) tested in our experiments did not allow detecting differences between disease subsets.

From a therapeutic standpoint, the interplay between IL-17A, TGF-β, and IL-6 is of major interest, since all these three cytokines are potential targets for therapy in SSc (42, 56–58). On the one hand, the direct antifibrotic role of IL-17A as well as its inhibitory activity on TGF-β-induced collagen production, would suggest that inhibition of this cytokine may have detrimental effects in humans affected by SSc. On the other hand, since IL-6 may directly or indirectly favor fibrosis, the blockade of factors enhancing the production of IL-6 may have favorable effects. In any case, therapeutic trials involving the blockade of any of these cytokines would profit of an in-depth analysis of their potential interactions.

## Ethics Statement

This study was approved by the ethical committee of the institutions involved (06-063, Commission cantonale d’éthique de la recherche, Geneva, Switzerland) and was conducted according to the Declaration of Helsinki. Written informed consent was obtained from each individual.

## Author Contributions

The author contribution was as followed: conceiving the research and manuscript drafting (AD and CC); performing the experiments (AD, MA, and BR); data analysis (AD, MA, and CC). All authors read and approved the final manuscript.

## Conflict of Interest Statement

The authors declare that the research was conducted in the absence of any commercial or financial relationships that could be construed as a potential conflict of interest.

## References

[1] Gabrielli A Avvedimento EV Krieg T Scleroderma. N Engl J Med (2009) 360(19):1989–2003. 10.1056/NEJMra0806188 19420368

[2] Varga JA Trojanowska M. Fibrosis in systemic sclerosis. Rheum Dis Clin North Am (2008) 34(1):115–43. 10.1016/j.rdc.2007.11.002 18329536 PMC9904084

[3] Allanore Y Simms R Distler O Trojanowska M Pope J Denton CP Systemic sclerosis. Nat Rev Dis Primers (2015) 1:15002. 10.1038/nrdp.2015.2 27189141

[4] Chizzolini C Brembilla NC Montanari E Truchetet ME. Fibrosis and immune dysregulation in systemic sclerosis. Autoimmun Rev (2011) 10(5):276–81. 10.1016/j.autrev.2010.09.016 20863906

[5] Wynn TA Ramalingam TR. Mechanisms of fibrosis: therapeutic translation for fibrotic disease. Nat Med (2012) 18(7):1028–40. 10.1038/nm.2807 22772564 PMC3405917

[6] Brembilla NC Chizzolini C T cell abnormalities in systemic sclerosis with a focus on Th17 cells. Eur Cytokine Netw (2012) 23(4):128–39. 10.1684/ecn.2013.0325 23360781

[7] Lafyatis R Transforming growth factor beta – at the centre of systemic sclerosis. Nat Rev Rheumatol (2014) 10(12):706–19. 10.1038/nrrheum.2014.137 25136781

[8] Khan K Xu S Nihtyanova S Derrett-Smith E Abraham D Denton CP Clinical and pathological significance of interleukin 6 overexpression in systemic sclerosis. Ann Rheum Dis (2012) 71(7):1235–42. 10.1136/annrheumdis-2011-200955 22586157

[9] Kawaguchi Y Contribution of interleukin-6 to the pathogenesis of systemic sclerosis. J Scleroderma Relat (2017) 2:S6–12. 10.5301/jsrd.5000258

[10] Bhattacharyya S Midwood KS Yin H Varga J. Toll-like receptor-4 signaling drives persistent fibroblast activation and prevents fibrosis resolution in scleroderma. Adv Wound Care (New Rochelle) (2017) 6(10):356–69. 10.1089/wound.2017.0732 29062592 PMC5649394

[11] Veldhoen M. Interleukin 17 is a chief orchestrator of immunity. Nat Immunol (2017) 18(6):612–21. 10.1038/ni.3742 28518156

[12] Kurasawa K Hirose K Sano H Endo H Shinkai H Nawata Y Increased interleukin-17 production in patients with systemic sclerosis. Arthritis Rheum (2000) 43(11):2455–63. 10.1002/1529-0131(200011)43:11<2455::AID-ANR12>3.0.CO;2-K 11083268

[13] Meloni F Solari N Cavagna L Morosini M Montecucco CM Fietta AM. Frequency of Th1, Th2 and Th17 producing T lymphocytes in bronchoalveolar lavage of patients with systemic sclerosis. Clin Exp Rheumatol (2009) 27(5):765–72. 19917158

[14] Murata M Fujimoto M Matsushita T Hamaguchi Y Hasegawa M Takehara K Clinical association of serum interleukin-17 levels in systemic sclerosis: is systemic sclerosis a Th17 disease? J Dermatol Sci (2008) 50(3):240–2. 10.1016/j.jdermsci.2008.01.001 18329249

[15] Radstake TR van Bon L Broen J Hussiani A Hesselstrand R Wuttge DM The pronounced Th17 profile in systemic sclerosis (SSc) together with intracellular expression of TGFbeta and IFNgamma distinguishes SSc phenotypes. PLoS One (2009) 4(6):e5903. 10.1371/journal.pone.0005903 19536281 PMC2691991

[16] Hsu E Shi H Jordan RM Lyons-Weiler J Pilewski JM Feghali-Bostwick CA. Lung tissues in patients with systemic sclerosis have gene expression patterns unique to pulmonary fibrosis and pulmonary hypertension. Arthritis Rheum (2011) 63(3):783–94. 10.1002/art.30159 21360508 PMC3139818

[17] Truchetet ME Brembilla NC Montanari E Allanore Y Chizzolini C. Increased frequency of circulating Th22 in addition to Th17 and Th2 lymphocytes in systemic sclerosis: association with interstitial lung disease. Arthritis Res Ther (2011) 13(5):R166. 10.1186/ar3486 21996293 PMC3308100

[18] Truchetet ME Brembilla NC Montanari E Lonati P Raschi E Zeni S Interleukin-17A+ cell counts are increased in systemic sclerosis skin and their number is inversely correlated with the extent of skin involvement. Arthritis Rheum (2013) 65(5):1347–56. 10.1002/art.37860 23335253

[19] Olewicz-Gawlik A Danczak-Pazdrowska A Kuznar-Kaminska B Gornowicz-Porowska J Katulska K Trzybulska D Interleukin-17 and interleukin-23: importance in the pathogenesis of lung impairment in patients with systemic sclerosis. Int J Rheum Dis (2014) 17(6):664–70. 10.1111/1756-185X.12290 24467649

[20] Gourh P Arnett FC Assassi S Tan FK Huang M Diekman L Plasma cytokine profiles in systemic sclerosis: associations with autoantibody subsets and clinical manifestations. Arthritis Res Ther (2009) 11(5):R147. 10.1186/ar2821 19799786 PMC2787259

[21] Chizzolini C Dufour AM Brembilla NC. Is there a role for IL-17 in the pathogenesis of systemic sclerosis? Immunol Lett (2018) 195:61–7. 10.1016/j.imlet.2017.09.007 28919455

[22] Wilson MS Madala SK Ramalingam TR Gochuico BR Rosas IO Cheever AW Bleomycin and IL-1beta-mediated pulmonary fibrosis is IL-17A dependent. J Exp Med (2010) 207(3):535–52. 10.1084/jem.20092121 20176803 PMC2839145

[23] Mi S Li Z Yang HZ Liu H Wang JP Ma YG Blocking IL-17A promotes the resolution of pulmonary inflammation and fibrosis via TGF-beta1-dependent and -independent mechanisms. J Immunol (2011) 187(6):3003–14. 10.4049/jimmunol.1004081 21841134

[24] Nakashima T Jinnin M Yamane K Honda N Kajihara I Makino T Impaired IL-17 signaling pathway contributes to the increased collagen expression in scleroderma fibroblasts. J Immunol (2012) 188(8):3573–83. 10.4049/jimmunol.1100591 22403442

[25] Cortez DM Feldman MD Mummidi S Valente AJ Steffensen B Vincenti M IL-17 stimulates MMP-1 expression in primary human cardiac fibroblasts via p38 MAPK- and ERK1/2-dependent C/EBP-beta, NF-kappaB, and AP-1 activation. Am J Physiol Heart Circ Physiol (2007) 293(6):H3356–65. 10.1152/ajpheart.00928.2007 17921324

[26] Brembilla NC Montanari E Truchetet ME Raschi E Meroni P Chizzolini C. Th17 cells favor inflammatory responses while inhibiting type I collagen deposition by dermal fibroblasts: differential effects in healthy and systemic sclerosis fibroblasts. Arthritis Res Ther (2013) 15(5):R151. 10.1186/ar4334 24289089 PMC3979123

[27] Lonati PA Brembilla NC Montanari E Fontao L Gabrielli A Vettori S High IL-17E and low IL-17C dermal expression identifies a fibrosis-specific motif common to morphea and systemic sclerosis. PLoS One (2014) 9(8):e105008. 10.1371/journal.pone.0105008 25136988 PMC4138152

[28] Wipff PJ Hinz B. Integrins and the activation of latent transforming growth factor beta1 – an intimate relationship. Eur J Cell Biol (2008) 87(8–9):601–15. 10.1016/j.ejcb.2008.01.012 18342983

[29] Gruschwitz M Muller PU Sepp N Hofer E Fontana A Wick G. Transcription and expression of transforming growth factor type beta in the skin of progressive systemic sclerosis: a mediator of fibrosis? J Invest Dermatol (1990) 94(2):197–203. 10.1111/1523-1747.ep12874503 2299195

[30] Sfikakis PP McCune BK Tsokos M Aroni K Vayiopoulos G Tsokos GC. Immunohistological demonstration of transforming growth factor-beta isoforms in the skin of patients with systemic sclerosis. Clin Immunol Immunopathol (1993) 69(2):199–204. 10.1006/clin.1993.1170 8403557

[31] Higley H Persichitte K Chu S Waegell W Vancheeswaran R Black C. Immunocytochemical localization and serologic detection of transforming growth factor beta 1. Association with type I procollagen and inflammatory cell markers in diffuse and limited systemic sclerosis, morphea, and Raynaud’s phenomenon. Arthritis Rheum (1994) 37(2):278–88. 10.1002/art.1780370218 7510487

[32] Ozbilgin MK Inan S. The roles of transforming growth factor type beta3 (TGF-beta3) and mast cells in the pathogenesis of scleroderma. Clin Rheumatol (2003) 22(3):189–95. 10.1007/s10067-003-0706-5 14505209

[33] Sargent JL Milano A Bhattacharyya S Varga J Connolly MK Chang HY A TGFbeta-responsive gene signature is associated with a subset of diffuse scleroderma with increased disease severity. J Invest Dermatol (2010) 130(3):694–705. 10.1038/jid.2009.318 19812599 PMC3867816

[34] Rice LM Padilla CM McLaughlin SR Mathes A Ziemek J Goummih S Fresolimumab treatment decreases biomarkers and improves clinical symptoms in systemic sclerosis patients. J Clin Invest (2015) 125(7):2795–807. 10.1172/JCI77958 26098215 PMC4563675

[35] Choy E Rose-John S Interleukin-6 as a multifunctional regulator: inflammation, immune response, and fibrosis. J Scleroderma Relat (2017) 2:S1–5. 10.5301/jsrd.5000265

[36] Denton CP Ong VH Interleukin-6 and related proteins as biomarkers in systemic sclerosis. J Scleroderma Relat (2017) 2:S13–9. 10.5301/jsrd.5000266

[37] O’Reilly S Ciechomska M Cant R van Laar JM Interleukin-6 (IL-6) trans signaling drives a STAT3-dependent pathway that leads to hyperactive transforming growth factor-beta (TGF-beta) signaling promoting SMAD3 activation and fibrosis via Gremlin protein. J Biol Chem (2014) 289(14):9952–60. 10.1074/jbc.M113.545822 24550394 PMC3975039

[38] O’Reilly S Cant R Ciechomska M van Laar JM Interleukin-6: a new therapeutic target in systemic sclerosis? Clin Transl Immunol (2013) 2(4):e4 10.1038/cti.2013.2 PMC4232056 25505952

[39] Kitaba S Murota H Terao M Azukizawa H Terabe F Shima Y Blockade of interleukin-6 receptor alleviates disease in mouse model of scleroderma. Am J Pathol (2012) 180(1):165–76. 10.1016/j.ajpath.2011.09.013 22062222

[40] Desallais L Avouac J Frechet M Elhai M Ratsimandresy R Montes M Targeting IL-6 by both passive or active immunization strategies prevents bleomycin-induced skin fibrosis. Arthritis Res Ther (2014) 16(4):R157. 10.1186/ar4672 25059342 PMC4220089

[41] Fielding CA Jones GW McLoughlin RM McLeod L Hammond VJ Uceda J Interleukin-6 signaling drives fibrosis in unresolved inflammation. Immunity (2014) 40(1):40–50. 10.1016/j.immuni.2013.10.022 24412616 PMC3919204

[42] Khanna D Denton CP Jahreis A van Laar JM Frech TM Anderson ME Safety and efficacy of subcutaneous tocilizumab in adults with systemic sclerosis (faSScinate): a phase 2, randomised, controlled trial. Lancet (2016) 387(10038):2630–40. 10.1016/S0140-6736(16)00232-4 27156934

[43] van den Hoogen F Khanna D Fransen J Johnson SR Baron M Tyndall A 2013 classification criteria for systemic sclerosis: an American college of rheumatology/European league against rheumatism collaborative initiative. Ann Rheum Dis (2013) 72(11):1747–55. 10.1136/annrheumdis-2013-204424 24092682

[44] LeRoy EC Black C Fleischmajer R Jablonska S Krieg T Medsger TA Jr Scleroderma (systemic sclerosis): classification, subsets and pathogenesis. J Rheumatol (1988) 15(2):202–5. 3361530

[45] Fineschi S Reith W Guerne PA Dayer JM Chizzolini C. Proteasome blockade exerts an antifibrotic activity by coordinately down-regulating type I collagen and tissue inhibitor of metalloproteinase-1 and up-regulating metalloproteinase-1 production in human dermal fibroblasts. FASEB J (2006) 20(3):562–4. 10.1096/fj.05-4870fje 16410344

[46] Chabaud M Fossiez F Taupin JL Miossec P. Enhancing effect of IL-17 on IL-1-induced IL-6 and leukemia inhibitory factor production by rheumatoid arthritis synoviocytes and its regulation by Th2 cytokines. J Immunol (1998) 161(1):409–14. 9647250

[47] Elias JA Lentz V Cummings PJ. Transforming growth factor-beta regulation of IL-6 production by unstimulated and IL-1-stimulated human fibroblasts. J Immunol (1991) 146(10):3437–43. 2026873

[48] Eickelberg O Pansky A Mussmann R Bihl M Tamm M Hildebrand P Transforming growth factor-beta1 induces interleukin-6 expression via activating protein-1 consisting of JunD homodimers in primary human lung fibroblasts. J Biol Chem (1999) 274(18):12933–8. 10.1074/jbc.274.18.12933 10212284

[49] Piek E Heldin CH Ten Dijke P. Specificity, diversity, and regulation in TGF-beta superfamily signaling. FASEB J (1999) 13(15):2105–24. 10.1096/fasebj.13.15.2105 10593858

[50] Massague J Chen YG Controlling TGF-beta signaling. Genes Dev (2000) 14(6):627–44. 10.1101/gad.14.6.627 10733523

[51] Verrecchia F Mauviel A. Transforming growth factor-beta signaling through the Smad pathway: role in extracellular matrix gene expression and regulation. J Invest Dermatol (2002) 118(2):211–5. 10.1046/j.1523-1747.2002.01641.x 11841535

[52] Yamamoto T. Pathogenic role of CCL2/MCP-1 in scleroderma. Front Biosci (2008) 13:2686–95. 10.2741/2875 17981743

[53] Chakraborty D Sumova B Mallano T Chen CW Distler A Bergmann C Activation of STAT3 integrates common profibrotic pathways to promote fibroblast activation and tissue fibrosis. Nat Commun (2017) 8(1):1130. 10.1038/s41467-017-01236-6 29066712 PMC5654983

[54] Zhou L Ivanov II Spolski R Min R Shenderov K Egawa T IL-6 programs T(H)-17 cell differentiation by promoting sequential engagement of the IL-21 and IL-23 pathways. Nat Immunol (2007) 8(9):967–74. 10.1038/ni1488 17581537

[55] Zhang S The role of TGF-beta in Th17 differentiation. Immunology (2018). 10.1111/imm.12938

[56] Beringer A Noack M Miossec P. IL-17 in chronic inflammation: from discovery to targeting. Trends Mol Med (2016) 22(3):230–41. 10.1016/j.molmed.2016.01.001 26837266

[57] Khanna D Jahreis A Furst DE Tocilizumab treatment of patients with systemic sclerosis: clinical data. J Scleroderma Relat (2017) 2:S29–35. 10.5301/jsrd.5000267

[58] Gyorfi AH Matei AE Distler JHW Targeting TGF-beta signaling for the treatment of fibrosis. Matrix Biol (2018) 68-69:8–27. 10.1016/j.matbio.2017.12.016 29355590

